# A Qualitative Systematic Review of Healthcare Practitioners’ Experience of Workplace Violence

**DOI:** 10.21315/mjms2024.31.1.4

**Published:** 2024-02-28

**Authors:** Ezatul Alia Md Emary, Siti Roshaidai Mohd Arifin, Muhammad Zubir Yusof

**Affiliations:** 1 Emergency Department, Hospital Sultan Idris Shah Serdang, Selangor, Malaysia; 2 Department of Special Care Nursing, Kulliyah of Nursing, International Islamic University Malaysia, Pahang, Malaysia; 3 Department of Community Medicine, Kulliyah of Medicine, International Islamic University Malaysia, Pahang, Malaysia

**Keywords:** workplace violence, experience workplace, healthcare practitioners, hospitals, qualitative studies

## Abstract

Healthcare practitioners face significant risks of workplace violence due to various reasons such as hospital congestion, miscommunication, and aggressive behaviours of patients and relatives. Exposure to workplace violence may disrupt the workflow process and compromise patient care in healthcare facilities, ultimately affecting job performance, reducing job satisfaction, and negatively affecting the physical and mental health of healthcare practitioners. This study aimed to review all the published studies conducted on the experiences of workplace violence among healthcare practitioners. This study is a systematic review of qualitative studies. Data were collected through online databases including ScienceDirect, PubMed, MEDLINE and JSTOR were searched from the year 2015–2021. The inclusion criteria were: qualitative methods and mixed methods of data collection and analysis; studies that were carried out among healthcare practitioners who have been experience on workplace violence; scope of the primary studies included experience of workplace violence; and published in English/Malay in academic journal between 2015 and 2021. A total of 15 papers were included in the final analysis. The overall quality of the included papers was high. Of the 15 papers, 12 studies fully met the CASP criteria. The results of the 15 included studies were organised into the thematic groups of: i) verbal violence as the common workplace violence; ii) perceived causes of workplace violence and iii) seeking help. Across different countries, verbal violence was the most common type of workplace violence reported by healthcare practitioners. This review also identified that a lack of information, failure to meet patient expectations, and delayed treatment were the main contributing factors to workplace violence.

## Introduction

Workplace violence is a severe occupational hazard in the healthcare industry worldwide, affecting thousands of healthcare practitioners each year (1, 2). Workplace violence encompasses a spectrum of unacceptable behaviours ranging from threats and verbal abuse to physical assaults and homicides in the workplace (3). For example, workplace violence incidents can occur when staff members are abused, threatened, discriminated against or assaulted in circumstances related to their work, including when they travel to and from work. Such incidents can pose threats to their safety, health and well-being (4).

Healthcare practitioners face the significant risks of workplace violence (1, 5). The annual rate of violent victimisation among nurses and physicians is 8.1 and 10.1 per 1,000 workers, which is much higher than 5.1 for all occupations (6). Workplace violence often results from congestion in healthcare facilities, miscommunication and aggressive behaviours displayed by patients and relatives. For instance, long waiting times in the emergency department can lead to impatience among patients, making them irritable and disruptive, sometimes harming healthcare staff (7).

Exposure to workplace violence may disrupt the workflow and patient care in hospitals. In the literature, doctors who suffered workplace violence have been linked with poor job performance, decreased job satisfaction and suboptimal physical health (8). Similarly, healthcare practitioners’ mental health is affected by increased feelings of guilt, anger, anxiety and depression (9). Furthermore, exposure to violence can compromise work morale and the satisfaction derived from patient care (10).

Many studies have investigated the phenomenon of workplace violence among healthcare practitioners worldwide. While it is clear that the prevalence varies from country to country—for example, 67% in Italy, 75% in Australia and 4.6% in Hong Kong (11–13)—there is no consensus on the experiences of workplace violence among healthcare practitioners. Therefore, this qualitative systematic review aimed to understand the experiences of healthcare practitioners regarding workplace violence in Malaysia and other countries.

## Methods

This was a systematic review of qualitative studies, also known as qualitative synthesis. This review aimed to understand the experiences of workplace violence among healthcare practitioners. A qualitative synthesis is a collection of methods for systematically reviewing and integrating the findings of qualitative studies. This approach was chosen to review all available literature and combine the findings of primary research studies to generate a comprehensive overview of the available evidence on the experience of workplace violence among healthcare practitioners. This approach is also suitable for new researchers to improve their knowledge of the subject of interest so that they can develop new research ideas and acquire critical skills by synthesising the results from the existing literature (14).

Four online databases were searched between 2015 and 2021, including ScienceDirect, PubMed, MEDLINE and JSTOR. The inclusion criteria were as follows: qualitative methods and mixed methods of data collection and analysis; studies that were carried out among healthcare practitioners who experienced workplace violence; scope of the primary studies included experience of workplace violence; factors triggering workplace violence; reported and unreported cases; and articles published in English/Malay in academic journals between 2015 and 2021. Upon eligibility assessment, 202 papers were excluded because they were not primary studies, not related to workplace violence or not conducted among healthcare practitioners. Further assessment excluded 48 papers because they were not published between 2015 and 2021 and were neither in English nor Malay.

The quality and methodology of the selected articles were assessed using the Critical Appraisal Skills Programme (CASP) (15). The following inclusion and exclusion criteria were used to screen the studies (Table 1).

**Figure 1:**
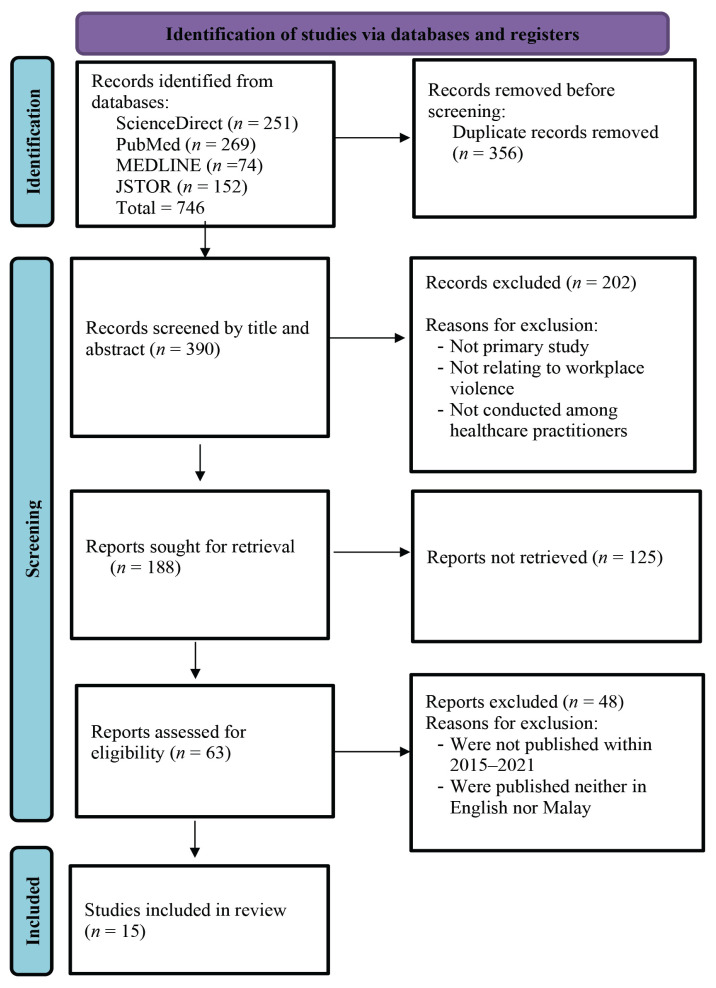
PRISMA flowchart

An overview of the research methodology using the Preferred Reporting Items for Systematic Reviews and Meta-Analyses (PRISMA) flowchart is presented in Figure 1.

### Qualitative Appraisal

Of the 15 studies, all but three fully met the CASP criteria, indicating that the overall quality of the included studies was high. This was derived from the CASP quality assessment (Table 2).

### Approach to Analysis

The selected papers were reviewed and analysed using thematic synthesis which is used to identify, analyse and interpret the patterns of various themes (31). Thematic analysis can be used when working as a research team to analyse large qualitative datasets. It is a step-by-step approach that provides a detailed explanation and practical approach for conducting thematic analysis. Thematic analysis is also useful in summarising the key features of large datasets by ensuring that researchers adopt a well-structured approach to deal with data so that clear and well-organised final reports can be generated (32).

Thematic analysis began by coding the text findings and reading the article’s findings line-by-line. Subsequently, ‘descriptive themes’ were developed and compared among the selected articles. Next, the ‘descriptive themes’ were refined to introduce a higher level of abstraction. In this step, some initial themes were retained, whereas others were grouped at an abstract level as the analysis progressed.

## Results

Fifteen studies were included in the meta-analysis. These studies were conducted worldwide in Malaysia, Indonesia, Thailand, Saudi Arabia, India, Iran, Turkey, Australia, Sweden, Italy and the United States of America. Nine studies employed face-to-face in-depth interviews (IDI), two involved focus group discussions (FGD), two used a combination of IDI and FGD, and two involved open-ended surveys. Studies were conducted among various groups of healthcare practitioners, with nurses being the major group of participants. A summary of the included studies is presented in Table 3.

Based on thematic analysis, the results of the studies included in this review can be categorised into the following three themes: i) verbal violence is the most common workplace violence, ii) perceived causes of workplace violence and iii) seeking help.

### Verbal Violence is the Most Common Workplace Violence

According to Fisekovic et al. (33), verbal abuse is a behaviour that shows humiliation, degradation or a lack of respect for a person’s dignity and worth. Analysis of the selected studies revealed that the most common type of workplace violence among healthcare practitioners was verbal violence (34). The abusive words reported in the studies included ‘prostitute’, ‘body fat’ and ‘husband snatcher’. Some were insulted through blackmail.

Henderson et al. (18) conducted a qualitative study by interviewing 19 nurses using snowball sampling to investigate their perspectives on violence caused by patients and visitors. The IDI lasted approximately 45 min–60 min. Nurses reported being verbally abused by patients and visitors, describing how they were shouted at, cursed at or sworn at. Additionally, Vrablik et al. (21) interviewed 23 healthcare practitioners, including nurses, advanced nurse practitioners, physicians and medical students, in three emergency departments in Washington. They reported that workplace violence occurred every day and felt that many people were loud and vocal toward healthcare practitioners. They also commented that workplace violence was a common phenomenon, noting that it was a ‘standard package’ that accompanied their jobs.

### Perceived Causes of Workplace Violence

Based on the 15 studies, workplace violence was perceived as a result of a lack of information, failure to meet patient expectations, refusal of unreasonable requests, delayed treatment/long waiting, poor management, workload pressure and a high number of visitors/patients.

Najafi et al. (29) conducted a qualitative study of workplace violence among 22 nurses in nine hospitals in Iran. Five categories of factors causing workplace violence were identified: i) unrealistic expectations from patients/relatives; ii) poor organisational management; iii) poor professional communication; iv) factors related to nurses; and v) factors related to patients, sick relatives and colleagues. In the category of unmet expectations of patients/relatives, one of the common triggers of workplace violence was patient-related; for example, when they received inadequate information or became anxious after finding out about the high treatment cost, especially in private hospitals.

Second, inefficient organisational management can cause workplace violence because of poor organisation, workload pressure or crowding (high patient and visitor volumes). One common example is how healthcare practitioners may not be able to handle patients and their caregivers effectively in overcrowded situations, a common scenario in emergency departments. Similarly, Kueanongkhun et al. (26) reported that workplace violence frequently occurred at the front desk of the emergency department because of the high number of patients and relatives there.

The third most commonly cited factor of workplace violence is inappropriate or non-professional communication, including verbal and nonverbal communication with patients/relatives or written reports in patient case notes. A similar study conducted in Italy by Ramacciati et al. (22) highlighted the role of weak communication among nurses in causing workplace violence. One participant said that nurses could trigger violence if they were hostile toward patients and visitors. Miscommunication in the workplace, whether between doctors and nurses or between nurses, can also indirectly result in workplace violence. Better communication between healthcare practitioners and patients/relatives can reduce anxiety and aggression.

Additionally, some participants reported that managers and doctors experienced violence and tension because of medical and nursing errors. Some nurses reported that communication between patients and relatives upon admission to the emergency department or other departments played a major role. In Turkey, Bahadir-Yilmaz and Kurşun (17) found that workplace violence encountered by nurses commonly arose due to poor communication skills, lack of information provided to patients, fatigue due to intense work pressure, job pressure and low job satisfaction. Most of these factors are closely associated with nurses’ responsibilities in patient care provision.

Finally, workplace violence can also be related to patient, relative or colleague factors. Patients who experienced pain or anxiety, hallucinations, restlessness, substance abuse (alcohol and drugs), had a history of suicide, or had an antisocial personality were more likely to commit workplace violence toward healthcare professionals. Some nurses pointed out that patients/relatives often become irritable because of the stress caused by their illness, consequently leading to tension and violence.

Nowrouzi-Kia et al. (35) conducted an IDI with 17 registered nurses from nine hospitals in Iran who had experienced workplace violence. The results showed that the factors contributing to workplace violence were lack of time to answer all questions from patients and relatives, insufficient information from doctors, and patients’ unrealistic expectations for nurses to fulfil all their demands.

### Seeking for Help

Although workplace violence is prevalent among health care practitioners, many cases remain unreported. Many seek comfort from their colleagues after the incident or dismiss the situation altogether, as they feel unsupported by the administrator. Some victims of workplace violence shared their experiences of seeking help after the incident.

In another study, 13 students from the medical, surgical and emergency departments of Shahid Beheshti Hospital in Kashan, Iran, were recruited using purposive sampling. One participant asked for help from hospital security when she encountered workplace violence, but they refused to get involved. Another participant claimed that the security team arrived only when the conflict had been settled (19).

In Malaysia, Salim et al. (24) conducted a study on workplace violence in the Emergency and Trauma Department of a tertiary government hospital. All healthcare practitioners with previous experience of workplace violence were interviewed. Incident reports were also reviewed. According to the participants, they considered themselves as being ‘immune’ to workplace violence and they believed that no help would be necessary. However, some sought help from colleagues to help them cope with their situation.

## Discussion

This review found that workplace violence has been reported across many countries, including Malaysia, Indonesia, Thailand, Saudi Arabia, India, Iran, Turkey, Australia, Sweden, Italy and the United States. The most commonly reported type of violence was verbal abuse, whereas the least common was physical violence. Factors triggering workplace violence included gender, patients under the influence of drugs and alcohol, communication challenges, long waiting times and overcrowded waiting rooms. According to Berkowitz (36), disinhibition occurs because of the lack of negative consequences following an aggressive act. In other words, apart from causing negative emotions, stress can lead to violence. Frustration is another unpleasant negative effect that can provoke violent actions in the workplace.

The three themes of workplace violence derived from the qualitative analysis were verbal violence as common workplace violence, perceived causes of workplace violence and seeking help. It is important to acknowledge that workplace violence should not be regarded as ‘part of the job’ (37). A safe, secure and productive work environment must always be maintained. Employers must take the necessary actions to minimise and prevent workplace violence. Moreover, specific policies to facilitate risk assessment and prevention should be implemented in the workplace to tackle this issue.

Most workplace violence was committed by patients and their relatives, with eight studies showing that the abuser was a patient and seven studies reflecting that the abusers were patients and relatives.

A recent study proposed recommendations to prevent workplace violence by increasing the number of security personnel and providing basic training to staff (38). According to the Occupational Safety and Healthcare Act (OSHA), policies and procedures must be put in place to prevent workplace violence, including the identification of risk factors and violent behaviour, comprehensive strategies for interacting with patients and dealing with violence, training programmes to equip employees with knowledge of operating safety equipment such as systems alarms, and ways of protecting themselves and colleagues during violent episodes (39).

## Conclusion

In summary, this study established three key findings. First, most violence experienced by healthcare practitioners at work is verbal. Second, the most commonly perceived causes of workplace violence included a lack of information, failure to meet patients’ expectations and long waiting times for treatment. Third, few qualitative studies on workplace violence among healthcare practitioners have been conducted in Malaysia. Therefore, further research is required to explore how Malaysian healthcare practitioners perceive their experiences of workplace violence to obtain more information about its impact on the Malaysian healthcare sector.

## Limitation

The limitation of the review was that there were few studies in Malaysia, especially qualitative studies. Therefore, the awareness of workplace violence in Malaysia remains low and needs to be emphasised to strengthen existing policies.

## Figures and Tables

**Table 1:** Inclusion and exclusion criteria of the study

Inclusion	Exclusion criteria
Qualitative method and mixed method of data collection and analysis. Peer review publication. Studies were carried out among healthcare practitioners with previous experience of workplace violence. The scope of the primary studies included the experience of workplace violence, factors that triggered workplace violence, reported and unreported cases. Published in English/Malay in academic journals between 2015 and 2021. Full paper available.	Review papers. Quantitative studies.

**Table 2:** Quality appraisal outcome using CASP

Author	Was there a clear statement of the aim of the research?	Is a qualitative methodology appropriate?	Was the research design appropriate to address the aims of the research?	Was the recruitment strategy appropriate to the aims of the research?	Was the data collected in a way that addressed the research issue?	Has the relationship between researcher and participants been adequately considered?	Have ethical issues been taken into consideration?	Was the data analysis sufficiently rigorous?	Is there a clear statement of findings?	How valuable is the research?
Zeighami et al. (16)	Yes	Yes	Yes	Yes	Yes	Yes	Yes	Yes	Yes	Yes
Bahadir-Yilmaz and Kursun (17)	Yes	Yes	Yes	Yes	Yes	Yes	Yes	Yes	Yes	Yes
Henderson et al. (18)	Yes	Yes	Yes	Yes	Yes	Yes	Yes	No	Yes	Yes
Al-Omari (19)	Yes	Yes	Yes	Yes	Yes	Yes	Yes	Unable to answer	Yes	Yes
Henderson et al. (20)	Yes	Yes	Yes	Yes	Yes	Yes	No	Yes	Yes	Yes
Vrablik et al. (21)	Yes	Yes	Yes	Yes	Yes	Yes	No	Yes	Yes	Yes
Ramacciati et al. (22)	Yes	Yes	Yes	Yes	Yes	Yes	Can’t answer	Yes	Yes	Yes
Beattie (23)	Yes	Yes	Yes	Yes	Yes	Yes	Yes	Yes	Yes	Yes
Salim et al. (24)	Yes	Yes	Yes	Yes	Yes	Yes	Yes	Yes	Yes	Yes
Salvador et al. (25)	Yes	Yes	Yes	Yes	Yes	Yes	Yes	Yes	Yes	Yes
Kueanongkhun et al. (26)	Yes	Yes	Yes	Yes	Yes	Yes	Yes	Yes	Yes	Yes
Yesilbas and Baykal (27)	Yes	Yes	Yes	Yes	Yes	Yes	Yes	Yes	Yes	Yes
Jakobsson et al. (28)	Yes	Yes	Yes	Yes	Yes	Yes	Yes	Yes	Yes	Yes
Najafi et al. (29)	Yes	Yes	Yes	Yes	Yes	Yes	Yes	Yes	Yes	Yes
Yosep et al. (30)	Yes	Yes	Yes	Yes	Yes	Yes	Yes	Yes	Yes	Yes

**Table 3:** Summary of the included studies

No.	Country	Author	Aims	Research design	Data collection	Qualitative analysis
1.	Malaysia	Salim et al. (24)	To examine the coping response to the occurrence of workplace violence encountered	Qualitative study	Semi-structured IDI with 13 healthcare practitioners working in emergency and trauma department	Thematic and content analysis
2.	Indonesia	Yosep et al. (30)	This study explored nurses’ perspective of work-related violence and traumatic experience related to workplace violence in Indonesia	Qualitative study	FGD with 40 nurses who are working in a referral mental health hospital	Qualitative data analysis (inductive and deductive category)
3.	Thailand	Kueanongkhun et al. (26)	To examine the prevalence of, perpetrators of, and factors associated with workplace violence among healthcare practitioners in tertiary hospitals	Mixed-method study	Qualitative data from 30 participants	Thematic analysis
4.	Saudi Arabia	Salvador et al. (25)	To explore the experiences of workplace violence among nurses	Descriptive phenomenology	IDI with 21 nurses	Colaizzi’s method
5.	India	Joshi and Joshi (20)	To understand the perceptions of young doctors on workplace violence	Qualitative study	Six individual face-to-face-IDIs and six FGDs with 41 young doctors	Thematic analysis
6.	Iran	Najafi et al. (29)	To explore Iranian nurses’ perceptions of and experiences with the antecedents and consequences of workplace violence perpetrated by patients, patients’ relatives, colleagues and superiors	Qualitative study	22 unstructured, IDIs were conducted with registered nurses who had experienced workplace violence in nine hospitals	Inductive content analysis
7.	Iran	Zeighami et al. (16)	To investigate the effects of sexual harassment in the workplace on Iranian nurses	Descriptive-explorative approach	Semi-structured interviews with 22 nurses	Conventional content analysis
8.	Iran	Al-Omari (19)	To explore experiences of workplace violence among nurses in medical, surgical, and emergency departments of a general hospital	Qualitative study	Semi-structured interviews among 13 nurses who had the experience of workplace violence	Content analysis
9.	Turkey	Yesilbas, and Baykal (27)	To explore the causes of violence against nurses exercised by patients and/or their relatives in different departments of Turkish hospitals	Qualitative descriptive design.	Semi-structured IDIs with 34 nurses working in different positions and departments from five different hospitals	Content analysis
10.	Turkey	Bahadir-Yilmaz and Kurşun (17)	The aim of this study was to assess the opinions of staff working in workplace-violence related units on violence against nurses	Qualitative and descriptive design	Face-to-face interviews with nine staff working in workplace-violence-related units in a research and training hospital	Qualitative content analysis
11.	Australia	Beattie et al. (23)	To examine the relationship between workplace violence perpetrated by clients, their innate neurophysiological response to disease, and the resulting interactions with healthcare providers	Explanatory study	Individual and group interviews with 99 managers, directors, health/safety staff, nurses and educators	Thematic analysis
12.	Sweden	Jakobsson et al. (28)	To explore how healthcare professionals in surgical hospital wards experience and manage workplace violence perpetrated by patients or visitors	Qualitative study	FGD interviews with 16 healthcare professionals working in surgical wards	Data were analysed using a thematic analysis
13.	Italy	Ramacciati et. al (22)	To explore the emergency nurse perceptions of workplace violence	Online open-ended survey	265 emergency nurses	van Kaan’s method
14.	The United States of America	Henderson et al. (18)	To investigate nurses’ perspectives on patient and visitor violence	Phenomenological design	Interview with 19 registered nurses	Colaizzi method
15.	The United States of America	Vrablick et al. (21)	To understand why workplace violence has a variable impact on individual healthcare workers	Phenomenological study	Interview with 23 emergency department healthcare workers who experienced a workplace violence	Content analysis and thematic analysis
